# Supplementary material for: A fault-intrusion-tolerant system and deadline-aware algorithm for scheduling scientific workflow in the cloud
Source: PeerJ Comput Sci. 2021 Nov 2;7:e747. doi: 10.7717/peerj-cs.747 (PMC8576554; doi:10.7717/peerj-cs.747)
Supplement: Supplemental Information 6 [file peerj-cs-07-747-s006.zip › Myproject1.1/docs/org/cloudbus/cloudsim/network/datacenter/class-use/NetworkHost.html]

Uses of Class org.cloudbus.cloudsim.network.datacenter.NetworkHost


JavaScript is disabled on your browser.


- Overview
- Package
- Class
- Use
- Tree
- Deprecated
- Index
- Help

- Prev
- Next

- Frames
- No Frames

- All Classes

## Uses of Class org.cloudbus.cloudsim.network.datacenter.NetworkHost

- Packages that use NetworkHost

  | Package | Description |
  | --- | --- |
  |  |  |
  | --- | --- |
  | org.cloudbus.cloudsim.network.datacenter |  |
- - ### Uses of NetworkHost in org.cloudbus.cloudsim.network.datacenter

    Fields in org.cloudbus.cloudsim.network.datacenter with type parameters of type NetworkHost

    | Modifier and Type | Field and Description |
    | --- | --- |
    |  |  |
    | --- | --- |
    | `java.util.SortedMap<java.lang.Double,java.util.List<NetworkHost>>` | Switch.`fintimelistHost` |
    | `java.util.Map<java.lang.Integer,NetworkHost>` | Switch.`hostlist` |

    Methods in org.cloudbus.cloudsim.network.datacenter that return NetworkHost

    | Modifier and Type | Method and Description |
    | --- | --- |
    |  |  |
    | --- | --- |
    | `protected NetworkHost` | Switch.`getHostwithVM(int vmid)` |

    Methods in org.cloudbus.cloudsim.network.datacenter that return types with arguments of type NetworkHost

    | Modifier and Type | Method and Description |
    | --- | --- |
    |  |  |
    | --- | --- |
    | `protected java.util.List<NetworkHost>` | Switch.`getfreehostlist(int numhost)` |

    Methods in org.cloudbus.cloudsim.network.datacenter with parameters of type NetworkHost

    | Modifier and Type | Method and Description |
    | --- | --- |
    |  |  |
    | --- | --- |
    | `protected double` | NetworkVmAllocationPolicy.`getMaxUtilizationAfterAllocation(NetworkHost host, Vm vm)` |

- Overview
- Package
- Class
- Use
- Tree
- Deprecated
- Index
- Help

- Prev
- Next

- Frames
- No Frames

- All Classes
